# Effects of Water Quality and Post-Harvest Handling on Microbiological Contamination of Lettuce at Urban and Peri-Urban Locations of Ouagadougou, Burkina Faso

**DOI:** 10.3390/foods7120206

**Published:** 2018-12-16

**Authors:** Juliane Dao, Kathrin Stenchly, Oumar Traoré, Philip Amoah, Andreas Buerkert

**Affiliations:** 1 Organic Plant Production and Agroecosystems Research in the Tropics and Subtropics (OPATS), Universität Kassel, Steinstrasse 19, D-37213 Witzenhausen, Germany; dao@uni-kassel.de (J.D.); buerkert@uni-kassel.de (A.B.); 2 Laboratoire National de Santé Publique, 09 B.P. 24, Ouagadougou 09, Burkina Faso; marrou4@yahoo.fr; 3 International Water Management Institute (IWMI), West Africa Office, PMB CT 112 Cantonments, Accra, Ghana; P.AMOAH@CGIAR.ORG

**Keywords:** urban agriculture, microbiological contamination, lettuce, trade chain

## Abstract

Vegetable production in urban gardens of Ouagadougou contributes to food security, but water for irrigation is often of low quality. This is particularly acute if irrigation water is taken from wastewater polluted channels. This study aimed at (i) verifying to what degree irrigation water quality is correlated with contamination of lettuce with *Escherichia coli*, total coliforms, and *Salmonella* spp., and (ii) assessing effects of post-harvest handling on pathogen development during the trade chain. We tested pathogen removal efficiency on lettuce by applying post-harvest washing. Irrigation water of production areas in Ouagadougou (*n* = 10) showed a mean *E. coli* load of 2.1 × 10^5^ CFU 100 mL^−1^. In 60% of the cases, irrigation water did not meet the standards of the World Health Organization (WHO) for safe irrigation water, and in 30% of the cases, irrigation water was contaminated with *Salmonella* spp. Loads of total coliforms on lettuce leaves ranged from 2.9 × 10^3^ CFU g^−1^ to 1.3 × 10^6^ CFU g^−1^, while *E. coli* averaged 1.1 × 10^2^ CFU g^−1^. Results on post-harvest handling revealed that microbial loads increased along the trade chain. Overall, half of all lettuce samples (*n* = 60) were tested positively for *Salmonella* spp. The experiment showed that appropriate post-harvest handling could prevent the increase of total coliforms.

## 1. Introduction

The rapidly growing population of West African cities results in an increasing demand for agricultural products [1]. Urban and peri-urban agriculture (UPA) supplies cities with vegetables, especially after the rainy season, but in the long lean season, it restricted to places where sources of irrigation water are present [2]. During this period, UPA farmers rely on well water, wastewater polluted channels, or dams [3]. In Ouagadougou, concrete channels run through the city to drain it of water after heavy rainfalls. They carry a mixture of natural streams and human sewage. The proportion of natural stream water, rain water, and wastewater depends on the season. The use of wastewater for irrigation and vegetable washing brings pathogens to the fields and ultimately to the vegetables, resulting in food borne diseases (FBD). FBDs are a major cause of morbidity and mortality in the human population of many countries and comprise a broad group of illnesses caused by enteric pathogens, parasites, chemical contaminants, and biotoxins [4]. Africa faces the highest burden of FBD, whereby 70% are diarrhoeal diseases caused by *Salmonella* spp. and pathogenic strains of the faecal bacteria, *Escherichia coli*, as well as *Vibrio cholera* [5]. Faecal contamination is particularly severe in raw edible vegetables, such as lettuce (*Lactuca sativa* L.), which is highly demanded by urban residents as shown in a recent study [6,7]. In Ouagadougou, lettuce is exclusively produced within UPA systems of which 50% constitute open-space vegetable farms in non-built-up areas. This lettuce is sold by women on official urban markets, informal markets, along main streets, and in individual small street shops. Hence, the production as well as the trade of vegetables is commonly informal. Over the years, traders have developed different strategies to keep vegetables fresh under the hot climatic conditions governing the trade chain. Rinsing the lettuce with water on the farm, and during transport and storage until sale is one such strategy.

However, previous studies have shown that in West Africa, agricultural produce is strongly polluted with bacterial pathogens as a consequence of wastewater usage on farmers’ fields and in markets [8,9,10,11]. The development of pathogen loads along the trade chain and especially the effects of post-harvest handling on the contamination level were widely neglected although the microbial load on lettuce in particular heavily depends on postharvest handling by market women.

Hence, this study aimed at verifying to what degree irrigation water quality is correlated with contamination of lettuce with *E. coli* and *Salmonella* spp. The effects of post-harvest handling on microbial development along the trade chain were also assessed. To this end we tested pathogen removal efficiency on lettuce by performing post-harvest washing under experimental conditions and studied post-harvest handling strategies of 10 trading women in Ouagadougou from the field to the consumer.

## 2. Materials and Methods

### 2.1. Study Area

Our study was conducted in Ouagadougou, the capital of Burkina Faso, a landlocked country in West Africa. The city is located in the sub-Sahelian climate zone and faces a short unimodal rainy season lasting four months from the end of May to the end of September with a precipitation of 600–900 mm per year [12].

**Figure 1:**
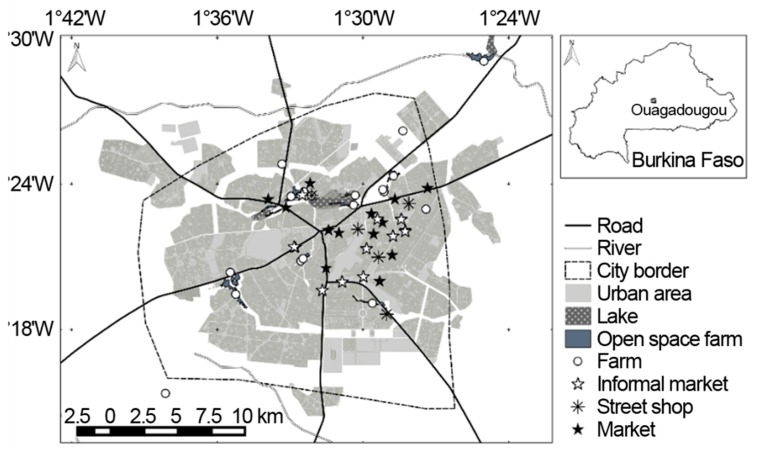
Location of the studied lettuce production farms (circle), in Ouagadougou (Burkina Faso) in 2014 with three different types of markets: Public market (black asterisk), informal market (white asterisk), and street shop (flake).

In total, 14 open-space systems where lettuce was cultivated were identified in the urban area of Ouagadougou, with 15 public markets, 10 informal markets, and five street shops as selling points (Figure 1).

### 2.2. Trader Interviews

From October 2013 to March 2014, 53 randomly selected traders who sold lettuce either on a public market, street market, or informal market in different locations were interviewed using a semi-structured questionnaire (Table S1). Key questions of the interview addressed the origin and final selling point of lettuce, times and regularity of trading activities as well as post-harvest handling regarding washing procedures and sources of washing water. The interviews took place directly at the farm site where traders purchased the lettuce from the producer or at the point of sale. Interviews were translated to the respective local language by an interpreter on site.

### 2.3. Microbial Contamination of Lettuce Leaves by Irrigation Water and Post-Harvest Handling

#### 2.3.1. Monitoring

To examine the development of microbial load on lettuce leaves induced by the quality of water used for irrigation and washing, 10 market women were accompanied from lettuce harvest to market place. Based on the interviews, 10 farming sites that differed in their irrigation water quality were selected. Traders were further interviewed in depth using open, reflective questions. All activities of market women that were related to lettuce and trading activities were recorded. Geographic coordinates from each farm site, transport route, and point of sale were collected. During the monitoring procedure in June and July 2014, a total of 60 lettuce samples were taken. The mean temperature was 29 °C and two rain events occurred during the sampling period. Each sample consisted of three lettuce heads without roots. Samples were collected by hand using gloves and placed in a sterile plastic bag. Two samples were taken at each time and location, meaning six lettuce heads per time point. Six of the 10 traders obtained the lettuce from farms where well water was used for irrigation and four traders from farms where polluted channel water was used for irrigation. The lettuce samples of each trader were taken at three time points during one day: At harvest, at market (arrival), and two hours after arrival. At each farm site, one water sample of five litres was taken from the irrigation water (*n* = 10). In case market women rinsed the lettuce before or during the selling process, samples of wash water were taken additionally (*n* = 9). To test irrigation water for counts of helminth eggs, 40 water samples, comprising two 1 l samples at each time and location, were taken and analysed separately. Sampling was repeated after two weeks.

#### 2.3.2. Post-Harvest Handling Experiment

To analyse the effect of appropriate post-harvest washing on microbial removal of lettuce leaves, two experimental fields with a size of 7 × 3 m each were cultivated in November and December 2014. One field was irrigated regularly with tap-water that showed a low total coliform load of 48 CFU 100 mL^−1^ when reaching the field. The second field was irrigated with channel water that measured a total coliform load of 6.3 × 10^4^ CFU 100 mL^−1^. This water was collected from the biggest channel of Ouagadougou, which is located at the outlet of the city and receives not only the river stream passing through the city forest, but also serves as a consolidation drainage avenue for all wastewater channels of the city.

At harvest stage, 18 lettuce heads were taken from both fields and separated into two batches. The first batch (*n* = 9) was kept unwashed for four hours and the second batch was washed directly after harvest with tap water. Three samples per batch, each consisting of three lettuce heads, were taken immediately after harvest and again after 2 h and after 4 h. Lettuce and water samples of the experiment were analysed for total coliform load as specified below. Samples were taken with gloves, sealed in polythene bags, transported on ice, and stored at a temperature of 4 °C until laboratory analysis within 24 h.

### 2.4. Laboratory Analysis

#### 2.4.1. Lettuce Microbiological Analysis

For laboratory analysis, 25 g each of the mixed lettuce sample was added to 225 mL of buffered peptone water (BPW, Liofilchem S.r.l., Teramo, Italy) and shaken gently by hand for two minutes. For the analyses of total coliforms and *E. coli*, dilutions (1 mL plus 9 mL diluent) until log six were performed and poured plates were done using 1 mL of the dilution before filling the fluid Chromocult ES agar (Merck KGaA, Darmstadt, Germany). Two dishes per dilution were incubated at 37 °C for total coliform and two dishes per dilution at 44 °C for the count of *E. coli*. The colonies were counted after 24 h, recounted after 48 h, and quantified in colony-forming units per gram of fresh material (CFU g FM^−1^), with the detection limits for both coliforms and *E. coli* being 10 CFU g FM^−1^.

The load of *Salmonella* spp. was determined following ISO 6579:2002. Pre-enrichment was done by using a stock solution with 25 g lettuce in 225 mL BPW. As a second solid selective plating-out medium, Salmonella Shigella Agar (SS, Oxoid Ltd., Hampshire, UK) was used. After the identification of sulfur-positive colonies on SS and Xylose lysine deoxycholate agar (XLD, Merck KGaA, Darmstadt, Germany), three suspected *Salmonella* spp. colonies per plate were confirmed by API 20E strips (BioMérieux, Lyon, France, [13,14]).

#### 2.4.2. Water Microbiological Analysis

Cellulose nitrate membrane filters with a pore size of 0.47 µm (Sartorius AG, Göttingen, Germany) were used in combination with a Sartorius Combisart^®^ system to filter the serial dilutions of the collected water samples. Filters were placed on the selective medium, Chromocult, to cultivate total coliforms and *E. coli*. To identify the counts of total coliforms, plates were incubated at 37 °C; for *E. coli*, the incubation temperature was 44°C. For *Salmonella* spp. identification, 2 L of the samples were filtered and the membranes were placed in 90 mL BPW for 24 h at 37 °C. Thereafter, 1 mL was taken from the pre-enrichment and added to 9 mL of the selective enrichment broth, Rappaport Vassiliadis Soya broth (RVS, Oxoid Ltd., Hampshire, UK), and incubated at 44 °C overnight. One µL of enriched broth was streaked onto the XLD agar (Oxoid Ltd., Hampshire, UK) and incubated at 37 °C for 24 h. Identification of the red colonies with a black centre was confirmed biochemically by API 20E strips [15]. To analyse water samples for the presence of *V. cholerae*, 2 L of water was filtered and membranes were enriched in alkaline peptone water (APW, Oxoid Ltd. Hampshire, UK) at 37 °C for 24 h. In case of very turbid water, more than one membrane was used and added to the enrichment broth. One ml of enriched broth was streaked onto the *Vibrio* selective agar, Thiosulfate Citrate Bile Salt Sucrose (TCBS, Merck KGaA, Darmstadt, Germany). Presumptive *V. cholerae* colonies on TCBS must be flat, circular, yellow, and sucrose-fermenting [16]. The presence of helminth eggs and larvae was analysed following Fulleborn’s flotation method [17].

### 2.5. Statistical Analysis

Statistical analysis of monitored data was performed by fitting a generalized linear mixed model (GLMM) with trader as random effect using Penalized Quasi-Likelihood (glmmPQL). The model tested if lettuce contamination depended on time, microbial load of irrigation water and the initial microbial load of lettuce (on-farm). The model was conducted using R version 3.2.3 [18], with additional functions provided by the R package MASS [19]. Effect of time, irrigation water source, and washing on the microbial load of lettuce (Post-harvest handling experiment) was tested using ANOVA after log-transformation of data. Statistical significance in differences of the mean was tested using post-hoc Least significant difference test (LSD test). ANOVA and LSD tests as well as visualizations were done with SPSS Version 24 (IBM Corp., Armonk, NY, USA) and Excel 2013 (Microsoft Corp., Redmond, WA, USA). The map was generated with Quantum GIS (Chugiak 2.4.0., QGIS Development Team 2012).

## 3. Results

### 3.1. Lettuce Trade Chain

The survey with lettuce traders (*n* = 53) showed that most of the lettuce was harvested in the morning (on average 6:30 a.m.) so that the markets could be reached by the official opening at 8 a.m. Some traders preferred to harvest in the afternoon, around 2 p.m., to sell on the market or on informal street markets at around 5 p.m., as this is the time when most people drive home and stop along their way to buy fresh vegetables for dinner. Occasionally, left over charges were sold in the morning on the markets. Older outer leaves were sold as cattle feed. Most of the interviewed lettuce traders sold their produce in markets (49%) and informal markets (34%), but also at individual street shops (17%). More than two thirds of the traders harvested the lettuce themselves; others bought the lettuce from resellers.

Nearly all traders (98%) washed the lettuce: either with tap or well water and often with both (Table 1). 42% of the traders responded that the consumers are interested in the origin of the lettuce, while 38% and 20% stated that consumers are not or only sometimes interested in the origin. Trading of lettuce was women’s domain, as all but one trader were female. They transported the lettuce in big baskets, mostly covered with a well water soaked cloth, on the back of small motorcycles or on bicycles to the selling point.

The monitoring of 10 lettuce traders yielded similar results as the survey: The majority of the traders harvested the lettuce in the morning and sold it on markets (Table 2 and Table S2). Those who harvested in the afternoon sold lettuce in street shops. In eight out of the 10 cases, the women washed the lettuce directly after harvest, mostly with well water. In half of the cases, women washed roots separately or took them off. Most of the lettuce was washed with tap water post-harvest. In all cases, the lettuce was washed at least once before being sold, in six cases, lettuce was even washed twice. Traders transported the lettuce over a distance of 0.05 km to 17.3 km, with an average distance of 8 km. Half of the observed lettuce charges reached the final sales location within 4 km and all lettuce heads were sold in less than 14 h.

### 3.2. Microbiological Contamination

#### 3.2.1. Relationship between Microbial Contamination of Lettuce and Irrigation Water Source

Water contamination levels of both irrigation water sources, wells, and channels, were similar with a wide range of *E. coli* from 1 × 10^2^ to 4.25 × 10^4^ CFU 100 mL^−1^ and of total coliforms from 3.6 × 10^3^ to 1.87 × 10^7^ CFU 100 mL^−1^. Two well water samples and one channel water sample were below 3 log units *E. coli* per 100 mL.

Microbiological contamination of lettuce on farms did not exceed 10^3^ *E. coli* counts per g FM lettuce and was neither related to the contamination of the irrigation water by *E. coli* (glmmPQL, *t* = −0.1, *p* = 0.9) nor to the time of harvest (*t* = 0.4, *p* = 0.7). In contrast, total coliform load on lettuce was positively related to the total coliform load of the irrigation water (*t* = 2.64, *p* = 0.04) and ranged between 2.89 × 10^3^ and 1.25 × 10^6^ CFU g FM^−1^. However, also here, harvest time did not effect changes in total coliform load on lettuce (*t* = −1.7, *p* = 0.14).

The presence of *Salmonella* spp. was detected within more than half of the tested irrigation water samples from wells (67%) and within one of the four tested channels. Two samples of irrigation water, both coming from wells, tested positively for helminth eggs of the species, *Strongylus* spp.

#### 3.2.2. Changes in Pathogen Load along the Trade Chain and the Effect of Post-Harvest Handling

**Figure 2:**
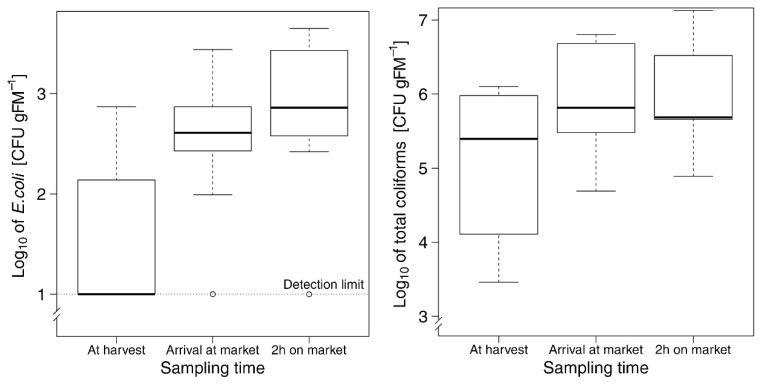
Load of *E. coli* (left) and total coliforms (right) on lettuce at the time of harvest, arrival at the market, and two hours after arrival in the market in Ouagadougou (Burkina Faso), 2014. Boxplots show the lower quartile, and median and upper quartile, with whiskers extending to the most extreme data point that is no more than 1.5 times the interquartile range from the edge of the box.

The contamination of lettuce through *E. coli* at the sales location ranged from below the detection limit of 10 CFU g FM^−1^ to 4.45 × 10^3^ CFU g FM^−1^ lettuce and was significantly higher than at the farm site (glmmPQL, *t* = 2.3, *p* = 0.03; Figure 2). The contamination of lettuce through *E. coli* increased further after two hours in the market (*t* = 4.1, *p* < 0.001). The documented post-harvest handling parameters, namely hours after harvest, number of washing events, distance to the market, as well as *E. coli* load of the wash water, did not affect the *E. coli* load on lettuce at the sales location (Table 3).

In contrast, total coliform load at the sales location showed a strong correlation to the initial total coliform load directly after harvest (Table 3), but was neither affected by hours after harvest, number of washing events, nor by distance to the market. Total coliforms ranged from 4.95 × 10^4^ to 1.35 × 10^7^ CFU g FM^−1^ (Figure 2) and its load increased with the length of time the lettuce remained in the market (*t* = 3.23, *p* = 0.002).

#### 3.2.3. Wash Water Quality

Tap water was transported to the location where lettuce got washed. Before washing, tap water showed a low contamination by *E. coli* (0.5 CFU 100 mL^−1^) and by total coliforms (117 CFU 100 mL^−1^) as well as no contamination with *Salmonella* spp. or *V. cholera*. However, if tap water was used for washing, total coliform reached 5 log CFU 100 mL^−1^ and *E. coli* 3 log CFU 100 mL^−1^. The use of tap-originated water for washing at the sales location tended to decrease the load of *E. coli* on lettuce, but not significantly (glmmPQL, *t* = −1.51, *p* = 0.17, Table 3).

Irrespective of irrigation water source or washing procedure, nearly 20% of the water samples and half of the lettuce samples were tested positively for *Salmonella* spp. One channel water sample was tested positively for *Salmonella* spp., as well as two well water samples and one wash water sample.

Overall, 62.5% of samples that were washed with channel or well water, 50% of the unwashed and 20% of the tap water washed samples were positively tested for *Salmonella* spp. All water samples that tested negatively for *V. cholerae* lettuce samples were excluded from examination for contamination.

#### 3.2.4. Effects of Appropriate Post-Harvest Lettuce Handling on Total Coliform Load under Controlled Conditions

Results gathered from our experiment under controlled conditions showed a significant effect of irrigation water source (tap or channel water, *F* = 62.4, df = 1, *p* < 0.001), washing procedure (washed or not washed, *F* = 54.5, df = 1, *p* < 0.001), and time after harvest (two and four hours, *F* = 15.8, df = 2, *p* < 0.001) on total coliform load of lettuce.

**Figure 3:**
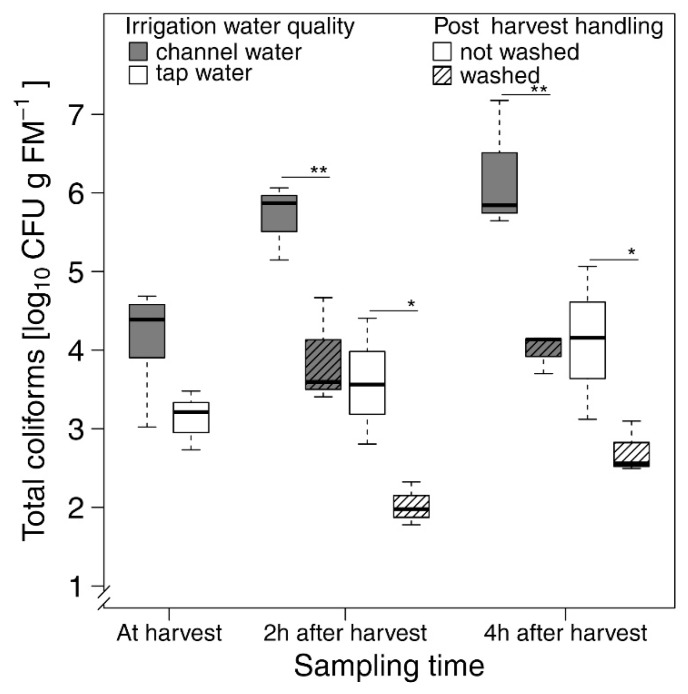
Experiment to evaluate the effect of irrigation water source (channel and tap water) and post-harvest washing on the total coliform load of lettuce cultivated in an urban production farm in Ouagadougou (Burkina Faso) in 2014. Stars indicate significant differences between the mean of washed and unwashed samples using *p* < 0.01 (*) and *p* < 0.001 (**) as significance thresholds. Boxplots show the lower, median, and upper quartile, with whiskers extending to the most extreme data point that is no more than 1.5 times the interquartile range from the edge of the box.

Total coliform contamination of lettuce irrigated permanently with tap water had 1.6 × 10^3^ CFU g FM^−1^ at harvest and was significantly lower than that of lettuce irrigated with channel water with 2.4 × 10^4^ CFU g FM^−1^ on average (LSD, *p* < 0.01; Figure 3). The difference between total coliform load on tap and channel water irrigated unwashed lettuce was still significant at later time points (after two and four hours; LSD, *p* < 0.001).

Without washing after harvest, total coliforms on channel-water irrigated lettuce increased significantly after two hours (LSD, *p* < 0.001), whereas this effect was not significant in tap water irrigated lettuce.

Results of the experiment showed further that the post-harvest washing of lettuce with tap water effectively limited the growth of coliforms during storage. This effect was more pronounced on lettuce plants irrigated with tap water for which post-harvest washing resulted even in a significantly lower total coliform load (2 h after harvest) than at harvest.

Channel water irrigated lettuce, which was washed with tap water after harvest, showed a significantly lower total coliform load after 2 and 4 h compared to unwashed samples (Figure 3). In tap-water irrigated lettuce, the washing effect was less, but still significant. Lettuce that was irrigated with channel water and washed with tap water had a constant amount of total coliform, whereas total coliforms on unwashed lettuce increased after 2 h and 4 h. In tap water, irrigated lettuce washing reduced the total coliform load significantly.

## 4. Discussion

### 4.1. Typology of Urban Traders

In Ouagadougou, the lettuce trade seems largely informal and is only partly regulated by governmental institutions at the official urban markets. Food trade of West African cities is often a women’s domain, as has been reported earlier [20], and run by individuals that are vulnerable to eviction [21]. In Ouagadougou, traders operated independently from associations documented in Ghana [22]. Contrary to Robineau’s [23] findings from Bobo Dioulasso, the second biggest city of Burkina Faso, in or study lettuce trading women were not married to lettuce farmers. In the capital, women mainly harvested the lettuce and sold it at their own shop or they were wholesalers who harvested and transported the lettuce to sell it to other traders. Still, personal networks were important, as reported by Porter et al. [20], because a particular lettuce field may belong to a family or friend and determines where the women harvested the lettuce. This is one reason why up to 30% of the trading women chose to gather the lettuce from farms that were located on the opposite side of the city to transport the fresh vegetables through the crowded city center to reach their selling point.

Nevertheless, transportation distances of lettuce to urban markets were relatively short as the highly populated city area does not exceed a 20 km diameter and lettuce is exclusively produced in urban and peri-urban open-space systems in close proximity to the inner city area.

Overall the survey and monitoring documented how individual traders were managing the lettuce trade, including details about irrigation quality, washing practice, selling locations, transport as well as harvesting and selling time. The complexity of lettuce post-harvest handling and possible contamination sources during their daily routine could only be detected through the use of qualitative methods.

The monitoring indicated that it was common to first wash lettuce directly on the farm. Afterwards, traders who remained in one location over many hours presented a small tap water washed portion on their stand and left the rest of the batch in a covered basket or bowl under the table. If more than one water source for washing was available, traders chose the one which appeared to be cleaner even if they had to pay for it. Mostly, it was well water on the farm, which was preferred to channel water and tap water in the market, and preferred to well water.

As already highlighted by Smit [21], the availability of tap water in markets—an infrastructure provided by the government- results in improved vegetable quality as the use of tap water reduced lettuce contamination. The choice of better quality wash water indicated the awareness of the trading women about contamination of water. In the in-depth interviews, as well as in the survey, women explained that farming areas, where polluted channel water was used for irrigation, were not favored for lettuce harvest. For the same reasons, consumers asked about the origin of the lettuce. All interviewees knew that lettuce had to be carefully washed before consumption. In contrast to Qadir et al. [24], these results show that consumer awareness of produce contamination was widespread in Ouagadougou. Still, producers, traders, and consumers may find it difficult to trust information about the crop trade chain, as trading is not regulated by policies and reliability of information depends on individual willingness and honesty. In addition to the contamination risks that traders were aware of, unconscious contamination risks were observed. For example, there were traders who washed lettuce with water used prior for babies’ personal care or allowed free-running poultry to have contact with the produce. Often selling points were dirty and under these conditions the placement of the perforated lettuce baskets on the floor seems to be particularly inadequate.

### 4.2. Relationship between Irrigation Water Quality and Pathogen Load on Lettuce Leaves

Only three out of the 10 water sources that were used for irrigation of lettuce in Ouagadougou were below the target threshold of WHO, which restricts irrigation water for labour intensive and raw edible crops to 3 log units of *E. coli* per 100 mL [25]. Urban channels in Ouagadougou drain combined water from rain, grey, black, or industrial effluents, and therefore contamination varies greatly depending not only on dilution effects, but also on location and season, as described. The studied wells in Ouagadougou were similarly contaminated with faecal bacteria, as reported earlier from rural and urban West African wells [26,27,28]. The bacterial contamination of well water through pathogenic loaded runoff water is most likely caused by the widespread lack of sanitary infrastructure in West African cities [29], but also by the intensive application of manure on urban vegetable fields in Ouagadougou [3,5].

Contamination of irrigation water beyond the sanitation threshold was even found in a water basin that receives water from a modern solar pump connected to a borehole (*E. coli* of 3.9 × 10^4^ per 100 mL), as it is the case in a peri-urban village of Ouagadougou. Contamination may have been introduced in between pumping and storing the water uncovered in the for local farmers free access basin, as drillings are normally not contaminated with *E. coli* [30].

Even if lettuce was irrigated with inappropriate water, *E. coli* was not typically present on lettuce leaves. One reason might be that lettuce had not been irrigated that same day, so that due to the low survival rate of *E. coli* in dry conditions [31], as well as the additional deactivation of *E. coli* by sunlight [32], loads on plants decreased.

### 4.3. Effect of Post-Harvest Handling on Lettuce Contamination

Winfield and Groisman [31] described how *E. coli* can reproduce in tropical humid non-host conditions. Under favourable conditions, therefore, natural bacterial growth can lead to the observed increase of total coliform and *E. coli* load on lettuce along the trade chain, from the field to the end-consumer. Furthermore, vegetable traders in West Africa have limited facilities to cool produce [33] which would allow to slow down bacterial growth. However, low cost alternatives, such as sprinkling lettuce with water and covering the produce with plastic to prevent cross-contamination and to keep it fresh, can also foster bacterial growth [34,35].

Besides the initial contamination of lettuce and the increase due to favourable growing conditions, cross-contaminations commonly occur due to contact with soil, manure, free-running chickens, or even due to binding the leaves into bunches and exposing them to dirt and dust [36]. At least six traders tried to clean off the soil by washing roots or taking them off to prevent contamination from soil and manure. Still, it has to be taken into account that *E. coli* is able to enter and survive in plants [37], which can further increase bacterial load, but this was not investigated in this study. Sprinkling water, which is in our study identical with wash water, did not seem to be a source of cross-contamination. Furthermore, the number of washing events and transport distance from farm to market did not significantly affect lettuce contamination. Washing with tap water reduced the bacterial load, as tap water quality in Ouagadougou met the WHO standards. However, as contaminated lettuce gets washed, successive lettuce heads washed with the same water may suffer cross-contamination due to the transfer of pathogenic bacteria by the washing water [38]. *Salmonella* spp. is known to contaminate water and by this infect previously uncontaminated lettuce samples as it is long-term persistent in non-host environments [31,39,40]. In our study, *Salmonella* spp. contamination of lettuce was comparable to that studied by Traore et al. [15], as 50% of the lettuce samples taken in Ouagadougou were loaded with this pathogen. Cross-contamination by covering lettuce with plastic, older leaves, or wetted cloth could not be proven and is unfortunately also neglected by the literature.

To support our findings from the field work, the experiment, which excluded cross-contamination, showed that (i) irrigation with clean water can significantly reduce initial bacterial contamination and (ii) it is possible to reduce bacterial loads of lettuce by washing once with clean water. The cleansing effect of post-harvest washing of lettuce may be optimized by using additives to the wash water, such as chlorine solution, as reported by Amoah et al. [11] and O’Flaherty et al. [41].

## 5. Conclusions

Except for tap water, all water sources were contaminated with potential pathogenic microbes. In view of the normal practice of the trading women, (i) the use of irrigation water for washing did not sufficiently reduce or even increased microbial loads on lettuce, and (ii) washing with tap water reduced microbial loads, but the wash water must be changed more often in order to prevent pathogen transfer. Contamination pathways other than water, such as soil, personal hygiene of traders, free-running animals, contaminated transport material, dust, and dirt are of importance. If lettuce is handled adequately and washed with tap water after harvest, it is possible to keep microbial loads down even if the crop was irrigated with low quality wastewater.

## Figures and Tables

**Table 1:** Survey results about harvest time, sales location, washing practice, and water source of 53 interviewed urban and peri-urban lettuce traders in Ouagadougou (Burkina Faso), 2014.

Activity	Time		Selling Location		Wash Water Source				Total Washing Events	
		% (*n*)		% (*n*)	On Farm	% (*n*)	In Market	% (*n*)		% (*n*)
Harvest	Morning *	53 (28)	Official market	40 (21)	Well water	49 (26)	Well water	15 (8)	Washed twice	45 (24)
	Afternoon	4 (2)	Informal market	15 (8)	Not washed	28 (8)	Tap water	8 (4)	Not washed	4 (2)
	Morning & afternoon	11 (6)	Street shop	13 (7)						
Resale without harvesting		32 (17)	Informal market	19 (10)	No information of whether lettuce was washed on farm		Tap water	25 (13)	No information	
			Official market	9 (5)			Not washed	8 (4)		

* Morning hours from 5 a.m. to 9 a.m.

**Table 2:** Detailed monitoring results about post-harvest handling of 10 lettuce traders from harvest at urban and peri-urban gardens to their sales location in Ouagadougou (Burkina Faso), 2014.

Irrigation Water	Wash Water Source on Farm	Prewash of Roots	Trader ID	Harvest Time	Beginning of Sale	Sales Location	Washing Practice	Total Distance
Well	Well	Yes	T7	7 a.m.	8.15 a.m.	Official market	Small portions with used tap water	3.8 km
	Well	No	T3	8 a.m.	9.40 a.m.	Official market	Small portions with used tap water & sprinkled with wash water, wash water was used to wash all later	13.3 km
						*Change location*		
					5 p.m.	Informal market		
	Well	Yes	T4	10.30 a.m.	3 p.m.	Informal market	Washed with tap water	16.5 km
	Well	No	T9	9.30 a.m.	10.30 a.m.	Official market	Lettuce not washed, but wetted with tap water	14.2 km
Channel	Well	No	T2	8 a.m.	9 a.m.	Markets & houses	Lettuce not washed	11.8 km
						*Change trader*		
						Official market	Small portions with used tap water	17.3 km
	Well	No roots harvested	T10	6.30 a.m.	7.30 a.m.	Official market	Lettuce not washed,	1.7 km
						*Change trader*	Washed with tap water	
					5 p.m.	Street shop	Sprinkled with tap water	8.3 km
	Well	No	T5	8 a.m.	9 a.m.	Official market	Lettuce not washed	
						*Change trader*		
					11 a.m.	Official market	Washed with tap water	2.9 km
	Channel	Yes	T6	3 p.m.	3.30 p.m.	Street shop	Lettuce not washed	1.4 km
Well	Not washed	Yes	T8	3 p.m.	3.30 p.m.	Street shop	Washed with well water	0.1 km
		Yes	T1	8 a.m.	5 p.m.	Street shop	Washed with tap water	1.5 km

**Table 3:** Results from the glmmPQL model that was used to test for influencing factors on the contamination of lettuce by *Escherichia coli* and total coliforms at different locations and different sampling times during a monitoring of 10 lettuce traders in Ouagadougou (Burkina Faso), 2014. Significant relations between the contamination degree of lettuce and an explanatory variable are highlighted in bold using *p* = 0.05 as the significance threshold.

At Harvest (T1), on Farm		Arrival at Market (T2)		2 h in Market (T3)	
*Escherichia coli*					
Load irrigation water	*t*_6_ = −0.1, *p* = 0.9	Initial load (at T1)	*t*_9_ = −0.6, *p* = 0.6	Load at T2	*t*_9_ = −0.9, *p* = 0.4
Water source (well)	*t*_6_ = 1.2, *p* = 0.3	Hours after harvest	*t*_5_ = 1.2, *p* = 0.6	Hours after harvest	*t*_6_ = 0.02, *p* = 0.9
Harvest time	*t*_6_ = 0.4, *p* = 0.7	No. washing events	*t*_5_ = 0.4, *p* = 0.9	No. washing events	*t*_6_ = 0.04, *p* = 0.9
		Distance to market	*t*_5_ = 0.4, *p* = 0.4	Tap water usage	*t*_6_ = −1.5, *p* = 0.2
		Load wash water	*t*_5_ = 0.4, *p* = 0.7		
Total coliforms					
**Load irrigation water**	***t*_6_ = 2.6, *p* = 0.04**	**Initial load (at T1)**	***t*_9_ = 3.3, *p* = 0.01**	Load at T2	*t*_9_ = −0.5, *p* = 0.7
Water source (well)	*t*_6_ = 1.4, *p* = 0.2	Hours after harvest	*t*_5_ = 1.9, *p* = 0.1	Hours after harvest	*t*_6_ = −0.5, *p* = 0.6
Harvest time	*t*_6_ = −1.7, *p* = 0.1	No. washing events	*t*_5_ = 0.5, *p* = 0.6	No. washing events	*t*_6_ = −0.1, *p* = 0.9
		Distance to market	*t*_5_ = −1.5, *p* = 0.2	Tap water usage	*t*_6_ = −0.2, *p* = 0.8
		Load wash water	*t*_5_ = −0.3, *p* = 0.8		

## Supplementary Materials

The following are available online at http://www.mdpi.com/2304-8158/7/12/206/s1, Table S1: Collected data and questions posed during individual interviews in a survey to evaluate post-harvest handling strategies from trading women and vegetable seller on markets and streets in Ouagadougou, Burkina Faso during 2013 and 2014, Table S2: Results of monitoring the post-harvest handling of ten lettuce traders from harvest in urban and peri-urban gardens to their selling points in Ouagadougou, 2014.
