# Supplementary material for: Effects of Water Quality and Post-Harvest Handling on Microbiological Contamination of Lettuce at Urban and Peri-Urban Locations of Ouagadougou, Burkina Faso
Source: Foods. 2018 Dec 16;7(12):206. doi: 10.3390/foods7120206 (PMC6306915; doi:10.3390/foods7120206)
Supplement: Supplementary file 1 [file foods-07-00206-s001.pdf]

Supplementary material

Table S1 Collected data and questions posed during individual interviews in a survey to evaluate post-harvest handling strategies from trading women and vegetable seller on markets and streets in Ouagadougou, Burkina Faso during 2013 and 2014.

**Collected data and posed questions**	**Data type**
Which vegetables are often eaten raw (without cooking)?	Qualitative
Which of these vegetables do you sell?	Qualitative
Which season of the year is the main sales period of these vegetables?	Qualitative
What are your sources of supply?	Categorical: farm, market, others
From which farm/market do you obtain the vegetables? (Information on exact location via GPS tracking)	Qualitative (+ GPS coordinates)
At which time and in which intervals do you obtain the vegetables from farm/market?	Qualitative/Integer
Please describe the handling of the vegetables from the time of purchase on the farm/market and resale on the market. (Cutting, washing, etc.)	Qualitative
What's your sales location? (Name of the market, street, customer, etc.; Information on exact location via GPS tracking)	Qualitative (+ GPS coordinates)
On which days of the week and to which time are you selling the produce?	Integer/Categorical
Does consumer/buyer ask for the exact location of the farm where the vegetables were cultivated?	Binary, yes/no
Does consumer/buyer ask for the used irrigation practice on farm (wastewater usage)?	Binary, yes/no
In case of customer questions, how often are these questions asked?	Integer
Are you willing that we accompany you for a few days on your sales tour from farm to market?	Binary, yes/no

Table S2 Results of monitoring the post-harvest handling of ten lettuce traders from harvest in urban and peri-urban gardens to their selling points in Ouagadougou, 2014.

Irrigation water	Wash water source on farm	Prewash of roots	ID	Harvest time	Begin of sale	Location of sale	Type of lettuce cover	Washing practice	Transport by	Total distance (km)
Well	Well	Yes	T7	7:00 AM	8:15 AM	Official market	Old washed leaves and plastic sheet	Washed small portion with used tap water	Bicycle	3.75
Well	Well	No	T3	8:00 AM	9:40 AM<br>↓*Change sellining location*<br>5:00 PM	Official market<br><br>Informal market	Jute sack or cloth	Washed small portion with used tap water and sprinkled with wash water, later washed all with used water	Motorbike	13.3
Well	Well	Yes	T4	10:30 AM	3:00 PM	Informal market	Old washed leaves<br>Jute sack after washing	Wash all with tap water	Motorbike	16.5
Well	Well	No	T9	9:30 AM	10:30 AM	Official market	Washed plastic sheet	No post-harvest wash,<br>just wetens with tap water	Motorbike	14.2
Channel	Well	No	T2	8:00 AM	9:00 AM<br>↓*Change trader*	Markets & houses<br><br>Official market	Dry cloth<br><br>Not covered	No post-harvest wash<br><br>Washed small portion with used tap water	Motorbike	11.75<br><br>17.3
Channel	Well	No roots harvested	T10	6:30 AM	7:30 AM<br>↓*Change trader*<br>5:00 PM	Official market<br><br>Street shop	Old washed leaves and cloth<br><br>Not covered	No post-harvest wash<br>Wash with tap water at home and sprinkled with tap water	Motorbike<br><br>Bicycle	1.7<br><br>8.3
Channel	Well	No	T5	8:00 AM	9:00 AM<br>↓*Change trader*<br>11:00 AM	Official market<br><br>Official market	Well water wet cloth<br><br>Plastic sheed	No post-harvest wash<br><br>Washed two times with tap water	Motorbike	2.95
Channel	Channel	Yes	T6	3:00 PM	3:30 PM	Street shop	Old washed leaves and jute sack	No post-harvest wash	Bicycle	1.35
Well	Not washed	Yes	T8	3:00 PM	3:30 PM	Street shop	Not covered	Washed with well water	Walking	0.05
Well	Not washed	Yes	T1	8:00 AM	5:00 PM	Street shop	Old washed leaves and wet cloth	Washed at home with tap water	Bicycle	1.47
